# The Intracellular Localization of ID2 Expression Has a Predictive Value in Non Small Cell Lung Cancer

**DOI:** 10.1371/journal.pone.0004158

**Published:** 2009-01-08

**Authors:** Jérôme Rollin, Claire Bléchet, Sandra Régina, Arthur Tenenhaus, Serge Guyétant, Xavier Gidrol

**Affiliations:** 1 CEA, DSV, IRCM, Laboratoire d'Exploration Fonctionnelle des Génomes, Evry, France; 2 INSERM, U618, and IFR 135, Tours, France; 3 Laboratoire d'anatomie pathologique, Bretonneau Hospital, Tours, France; 4 Service d'Hématologie-Hémostase, Trousseau Hospital, Tours, France; University of Munich and Center of Integrated Protein Science, Germany

## Abstract

**Background:**

ID2 is a member of a subclass of transcription regulators belonging to the general bHLH (basic-helix-loop-helix) family of transcription factors. In normal cells, ID2 is responsible for regulating the balance between proliferation and differentiation. More recent studies have demonstrated that ID2 is involved in tumor progression in several cancer types such as prostate or breast.

**Methodology/Principal Findings:**

In this work, we investigated, for the first time, the relationship between the expression of ID2 in non-small cell lung cancer (NSCLC) patients and the clinicopathological features and prognosis of these patients. Immunohistochemistry was performed on tissue microarrays, which included 62 NSCLC tumors. In malignant tissues, ID2 expression has been detected in both the nuclear and cytoplasmic compartments, but we have demonstrated that only nuclear expression of ID2 is inversely correlated with the differentiation grade of the tumor (p = 0.007). Interestingly, among patients with poorly differentiated tumors, high nuclear expression of ID2 was an independent and unfavorable prognostic factor for survival (p = 0.036).

**Conclusions:**

These results suggest that ID2 could be involved in tumor dedifferentiation processes of NSCLC, and could be used as prognostic marker for patients with poorly differentiated tumors.

## Introduction

ID2 (Inhibitor of DNA binding 2) belongs to a subclass of proteins, the ID family, found within the large bHLH (basic-helix-loop-helix) group of transcription factors. Interestingly, as they do not possess the basic domain, ID proteins do not directly bind to DNA and, instead, act as dominant negative antagonists of class A bHLH transcription factors. Among the known targets of ID proteins are the E protein and members of the Ets family [1]. ID proteins are involved in tissue development [2], [3], and have been described as inhibitors of differentiation in numerous cell types including myeloid cells [4], B lymphocytes [5], and muscle cells [6]. ID2 expression is generally low in normal tissues, and is up-regulated in tumors. High levels of expression have been observed in malignancies such as neuroblastoma [7], [8], colorectal adenocarcinoma [9], Ewing sarcoma [10], prostate cancer [11], and pancreatic cancer [12]. Altogether, these results suggest that ID2 may play an important role in tumor initiation and progression.

To date, there is only a single study evaluating the role of ID2 in lung cancer, specifically in small cell lung cancer (SCLC) [13]. Lung cancer is a leading cause of deaths from cancer worldwide. The most abundant histological type that affects about 80% of patients is non-small cell lung cancer (NSCLC), as opposed to small cell lung cancer. The overall 5-year survival rate of NSCLC patients is about 15% [14]. Once a diagnosis has been established, treatment options are typically based on stage and histology [15], although this stratification scheme does not consistently reflect the biology of tumor. As a result, over 50% of patients with early-stage lung cancer will eventually die, while others at a more advanced stage will survive [14], [16], underlying the need for additional prognostic markers to better predict the outcome of the disease [17].

In this paper, we have evaluated the expression of ID2 by immunohistochemistry on NSCLC tissue microarrays containing 62 tumors. We then further investigated the relationships between ID2 expression and clinicopathologic parameters, in order to assess its possible role as a prognostic marker for NSCLC.

## Results

### ID2 Expression in Normal Lung and in NSCLC Tissues

**Figure 1:**
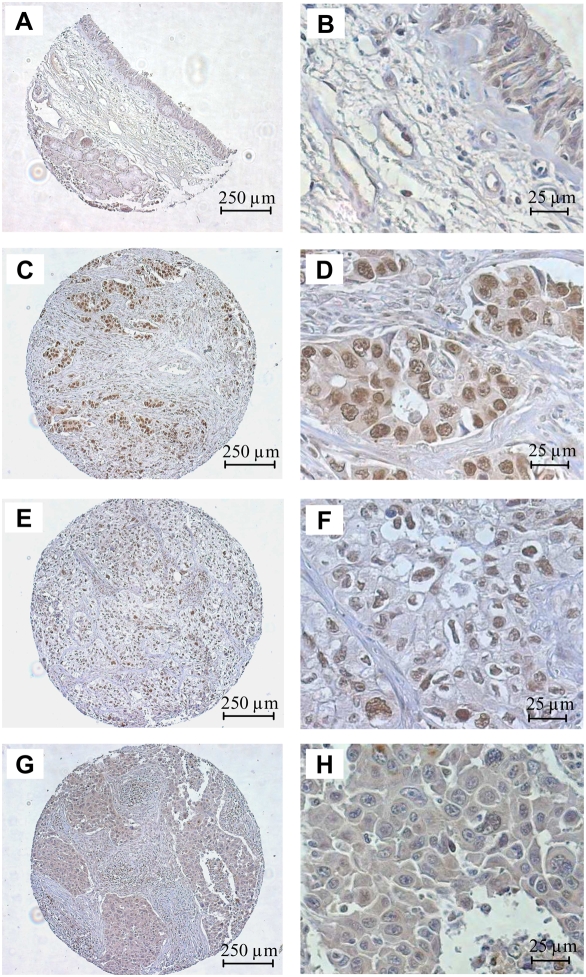
ID2 immunohistochemistry in normal bronchial epithelium (A–B) and in NSCLC (C–H). A–B: ID2 staining of epithelial and endothelial cells in non-cancerous lung. C–F: Representative expression on TMA with strong ID2 staining in nucleus and with moderate (C, D) or weak (E, F) cytoplasmic staining. C, D are poorly differentiated adenocarcinomas and E, F is a poorly differentiated large cell carcinoma. G–H: Tumor with absence of nuclear staining with moderate (G, H) cytoplasmic expression of ID2. G, H are moderately differentiated squamous cell carcinomas.

Immunostaining for ID2 was performed on a tumor microarray (TMA) containing 62 NSCLC samples from human patients. The clinical and histological features of patients are described in Table 1. In non-cancerous lung tissue, weak to moderate ID2 expression was detected in bronchial epithelial cells in both the cytoplasm and nuclei. In addition, weak ID2 staining was observed in the cytoplasm of endothelial cells (Figure 1A–D).

**Table 1:** Clinical and histological features of patients with NSCLC.

	n	(%)
**All**	62	100
**Sex**		
Female	8	13
Male	54	87
**Smoking status**		
Non smokers	5	8
Smokers	57	92
**Histological type**		
Adenocarcinoma	33	53
Squamous cell carcinoma	19	31
Other type	10	16
**Differentiation/Grade**		
Well	17	27
Moderately	26	42
Poorly	19	31
**T status**		
T1–T2	52	84
T3–T4	10	16
**N status**		
N0	38	62
N+	23	38
**M status**		
M0	52	84
M+	10	16
**Stage**		
I–II	36	58
III–IV	26	42

Other types: include large cell carcinoma, carcinoma, and bronchioalveolar carcinoma.

ID2 expression in tumor cells from the 62 NSCLC patients was highly diverse both in terms of intensity and localization (Figure 1). Out of 62 NSCLC patients, a large proportion (98%, 61/62) expressed ID2 protein almost exclusively in tumor cells (median of global staining score = 3; range 0 to 18). Nuclear immunoreactivity was positive in 33 (53.3%) of the NSCLC samples (median of nuclear staining score = 1; range 0 to 11), and cytoplasmic ID2 was detected in 57 (92%) out of 62 tumors (median of cytoplasmic staining score = 2; range 0 to 8). Many poorly differentiated tumors exhibited strong ID2 staining in the nuclei in conjunction with weak or moderate cytoplasmic staining (Figure 1E–J). In contrast, ID2 was not expressed in the nuclei of partially or well-differentiated tumors, while weak to moderate expression was observed in the cytoplasm (Figure 1K–P).

### Association of ID2 Expression with Clinicopathological Parameters

**Figure 2:**
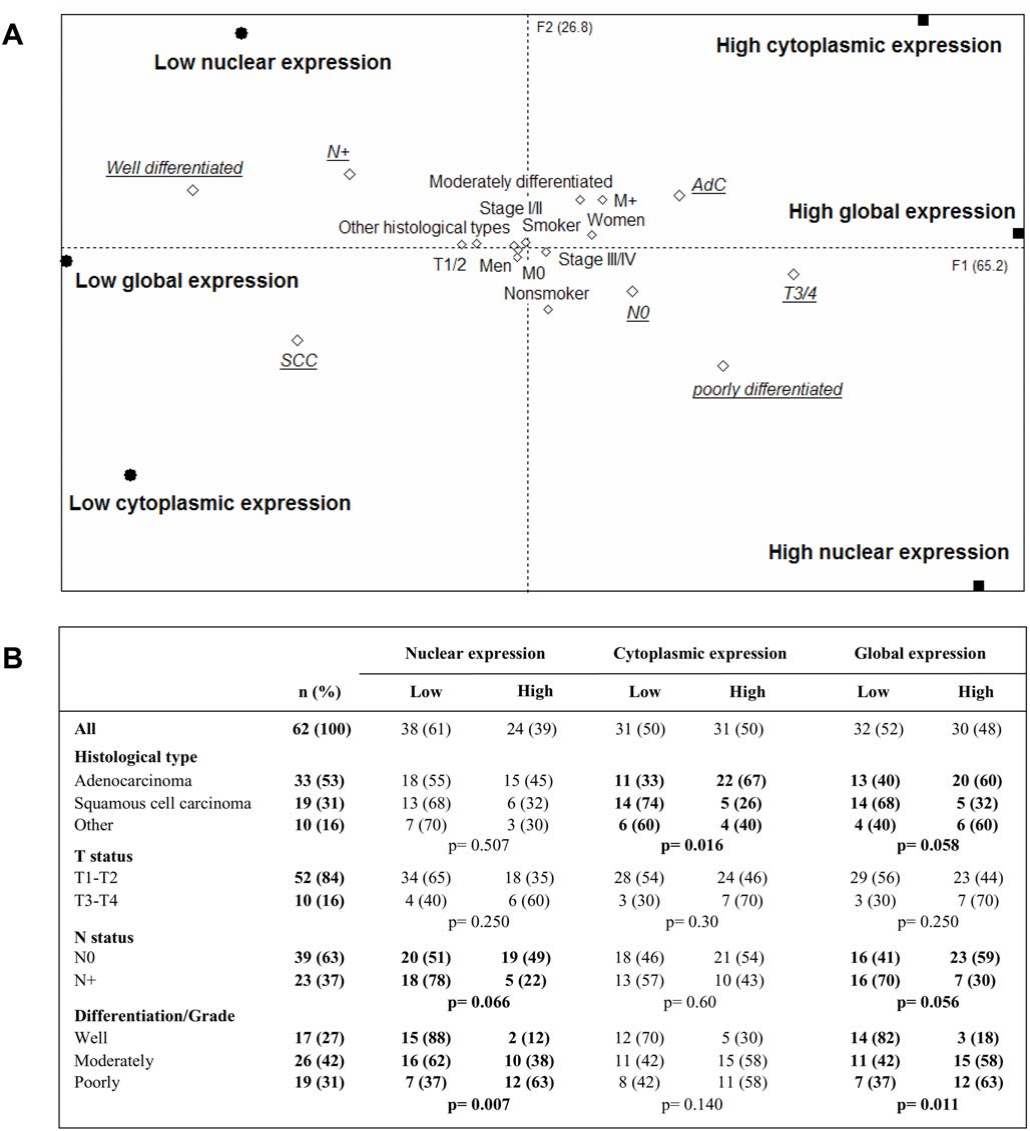
Graphic representation of multiple correspondence analysis (A) and DEMOD analysis results (B). A: Nuclear, cytoplasmic, and global expression of ID2 within NSCLC tumors have been used as active variables and are represented by bold squares and circle. Clinicopathological features (i.e., differentiation, histological type, genre, smoking status, stage, and TNM classification) have been used as illustrative variables and are represented by open lozenge. All variables were placed according to the gravity center of all patients belonging to the represented category. Clinical parameters potentially associated with ID2 expression are underlined and in italics. B: DEMOD analysis was performed to identify statistical associations between ID2 expression and clinicopathological features. Only parameters identified with MCA have been presented in the table (i.e., histological type, T status, lymph node involvement status, and differentiation of tumor).

The median of the staining score for either nuclear, cytoplasmic, or global expression of ID2, as described above, was used as a cut off to classify patients in two groups: “low” (i.e., low ID2 expression) and “high” (i.e., high ID2 expression). Multiple component analysis (MCA) was performed on data collected from the 62 patients and the graphical representation is presented in Figure 2A. In brief, variable neighborhoods on the graph are potentially associated together. MCA showed that low nuclear expression of ID2 was associated with well-differentiated tumors and lymph node involvement. In contrast, high expression of ID2 in the nucleus was seen more frequently in tumors that were poorly differentiated and had advanced local invasion (T3–T4 status). In association with MCA, DEMOD analysis was performed and demonstrated a significant association between nuclear expression of ID2 and histological differentiation of NSCLC (Figure 2B). Our results showed 15 out of 17 (88%) well-differentiated tumors exhibited low expression levels of ID2. In addition, poorly differentiated tumors had strong nuclear expression of ID2 (p = 0.007) in 12 out of 19 (63%) cases. However, no significant relationship was found between ID2 expression and lymph node involvement or T status, possibly due to the uneven distribution of T status between groups (52 and 10 patients in T1–T2 and T3–T4, respectively). Cytoplasmic expression of ID2 was not associated with the differentiation grade of the NSCLC, but was surprisingly linked to the histological type of tumors. Tumor expression of ID2 was high in 22 out of 33 (67%) cases of adenocarcinoma compared to 5 out of 19 (26%) cases of squamous cell carcinoma (p = 0.016) (Figure 2B).

### Univariate and Multivariate Survival Analysis

**Figure 3:**
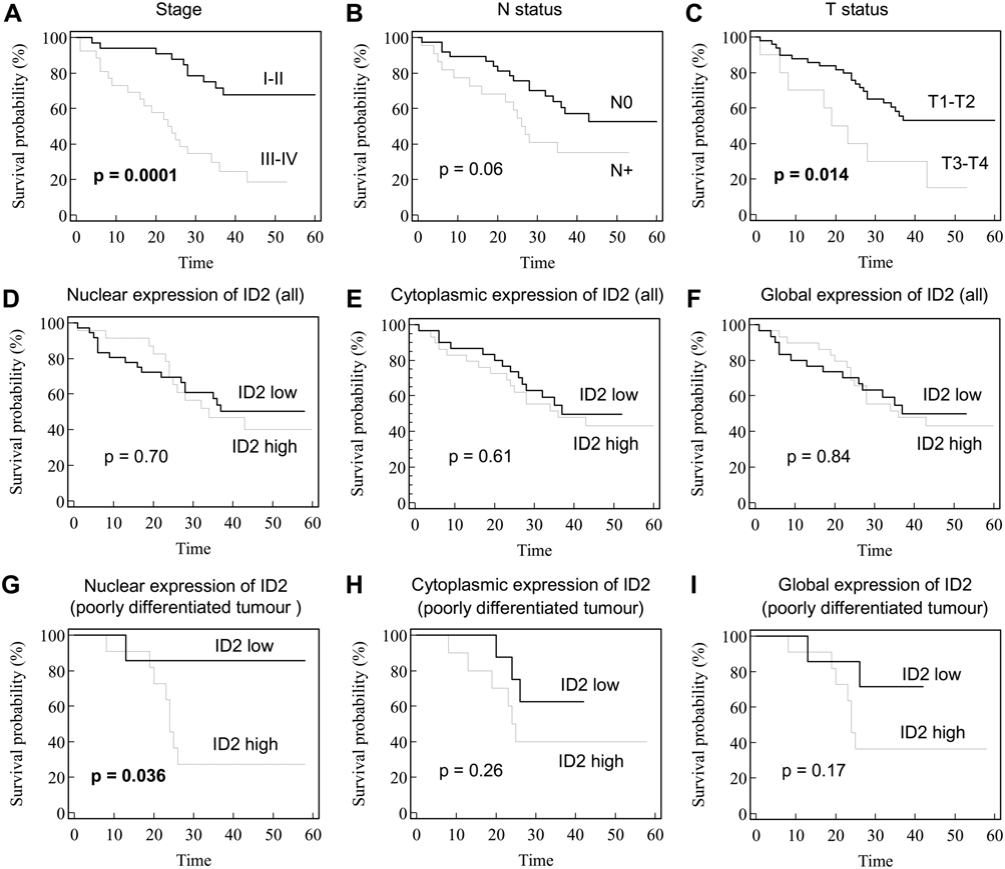
Survival analysis. A–F: Kaplan—Meier curves established for all NSCLC patients (n = 59) according to disease stage (A), nodal status (B), T status (C), nuclear (D), cytoplasmic (E), and global expression of ID2 (F). G–I: Kaplan—Meier curves obtained for patients with poorly differentiated tumors (n = 18) according to nuclear (G), cytoplasmic (H), and global (I) expression of ID2. Curves were compared with log-rank test.

To determine whether immunostaining of ID2 had a prognostic value, we examined the association between global, nuclear, and cytoplasmic expression of ID2 with the overall survival of patients. First, we analyzed conventional clinicopathological parameters. We observed a significant impact of tumor stage (p = 0.0001) and tumor size (p = 0.014) on patient overall survival (Figures 3A–C), whereas nodal status (N0 vs. N+) was of borderline significance (p = 0.06). Histological differentiation was not a prognostic marker in our cohort of NSCLC patients (p = 0.48; data not shown). Similarly, we did not observe a significant difference in the overall survival between patients with high expression levels of ID2 and those exhibiting low expression levels of ID2 (Figure 3D–E). However, after stratification of the data according to histological differentiation (Figure 3G–I), poorly differentiated NSCLC patients with high nuclear expression showed a shorter overall survival than patients with low ID2 nuclear expression (median of survival = 24 vs. 41 months, respectively; p = 0.036; Figure 3G). In addition, multivariate analysis using Cox's regression model demonstrated that nuclear ID2 expression in patients with poorly differentiated tumors was an independent prognostic factor of overall survival (p = 0.034; RR = 13.7; IC 95% = 1.2–161; Table 2). No prognostic value of ID2 nuclear expression was observed in moderate or well-differentiated tumors (data not shown).

**Table 2:** Mutivariate survival analysis after stratification according to patients with poorly differentiated tumors.

Variables	Relative Risk	95% CI	p value
T status	6.7	1.1–39.1	**0.035**
N status	2.05	0.4–9.5	0.36
Nuclear expression of ID2	13.7	1.2–161	**0.034**

## Discussion

The role of ID2 has been studied in several types of cancer (i.e., prostate, breast, colorectal, neuroblastoma, and very recently small cell lung cancer), but to date, nothing is known about the expression of ID2 in non-small cell lung cancer, which affects 80% of patients with lung malignancies. Here, we focused on the expression of ID2 in NSCLC and its relationship with the clinicopathological parameters and prognosis of the disease. Expression of ID2 was evaluated in 62 NSCLC tumors by immunohistochemistry using tissue microarrays. While the majority of the patients analyzed in this study expressed ID2, the patient group was very heterogeneous with regard to both expression level and intracellular localization. However, we have demonstrated that high ID2 expression in nuclei seems to be correlated with poor differentiation of the tumor. This observation is well in agreement with known properties of ID2, since the protein has been characterized as an inhibitor of differentiation in numerous tissues, including the epidermis and muscle [18], [19]. Thus, ID2 inhibits muscular differentiation by direct interaction with MyoD, a bHLH transcription factor required for myoblastic differentiation [20]. Moreover, in prostate carcinoma, strong ID2 expression was associated with poorly differentiated grades of tumor, suggesting that ID2 could also be involved in regulation of tumor dedifferentiation [11], [21]. Our data showed that a high nuclear expression level of ID2 was an independent and negative prognostic marker of survival in patients with poorly differentiated lung tumors. Thus, patients with tumors exhibiting high nuclear expression of ID2 have 13.7-fold higher risk of death than patients with low expression of ID2. The prognostic value of high ID2 expression has already been demonstrated in other cancer types such as neuroblastoma and prostate cancer [7], [11], [21], [22], [23]. However, previous studies on breast cancer and SCLC have reported that high cytoplasmic expression of ID2 was associated with a good prognosis, whereas no correlation was found between nuclear expression of ID2 and the survival of patients [13], [22]. Kamalian *et al.* suggested that in SCLC increased expression of ID2 in cytoplasm could be due to its export from nuclei and may reduce the pro-tumoral effect of ID2. These results were globally in agreement with our study, as we demonstrated a direct relationship between nuclear expression of ID2 and the survival of patients. However, differences regarding ID2 localization may originate in part from differences between the two pathologies, but also from differences in the specificity of the antibodies. Indeed, we have used Zymed antibodies as recommended by Perk *et al.*, who warned against the use of poorly specific ID2 antibodies [24]. Altogether, our data suggest that both ID2 expression and intracellular localization must be evaluated in order to determine the prognosis for cancer patients, including those with lung cancer. The intracellular localization of ID2 may play a major role in the control of ID2 function and cell fate regulation. Kurooka *et al.* determined that the nucleo-cytoplasmic shuttling of ID2 is an active mechanism dependent on the CRM-1 protein, and is inversely correlated to transcriptional repression via the E-box sequence [25]. In addition, Lasorella and Iavarone demonstrated that the interaction between ID2 and the actin-associated protein enigma homolog (ENH) also controls the localization of ID2 [26]. Indeed, downregulation of ENH expression prevents relocalization of ID2 into the cytoplasm in differentiated neuroblastoma cells. This result allows us to speculate on the possible implication of ENH or CRM-1 protein in the dedifferentiation mechanism of NSCLC. Our results indicate that nuclear ID2 is likely to be a prognostic factor in NSCLC and ENH-mediated translocation of ID2 to the cytoplasm may explain the lack of prognostic value for cytoplasmic ID2. However, the role of ID2 compartmentalization in lung tumor progression, although potentially interesting, would require further evaluation.

The mechanism by which ID2 promotes lung tumor progression is not clear yet. However, results obtained in other models suggest that the action of ID2 could depend on the inhibition of Rb tumor suppressor pathway [3], [27]. In addition, it has been demonstrated that ID2 also regulates angiogenesis and apoptosis via transcriptional regulation of the *VEGF* and *BCL2* genes, respectively [28], [29]. However, action of ID2 could also be tissue specific: in breast cancer cell lines, high expression of ID2 reduced growth potential and invasiveness, while up-regulation of ID2 increased proliferation and invasive potential in prostate tumor cells [11], [22], [23].

Finally, although our results are preliminary, they suggest that ID2 could be involved in the tumor dedifferentiation processes of NSCLC, and could be used as prognosis marker for patients with poorly differentiated tumors. Interestingly, in NSCLC, poorly differentiated carcinomas are difficult to classify, and as such, the identification of novel prognostic makers for this particular group may help improve the therapeutic outcome of the patients.

## Materials and Methods

### Tumor samples

We have analyzed tissue samples collected in a prospective group of 62 patients with NSCLC, who had undergone complete surgical resection of the lung tumor as an initial treatment (i.e., without prior radiotherapy or chemotherapy) between January 2002 and August 2004 at the Thoracic Surgery Department of Trousseau Hospital (Tours, France). Surgical specimens were fixed in 10% formalin and paraffin embedded for routine histological examination and immunohistochemistry. Histological types, differentiation, TNM classification, and stage grouping (summarized in Table I) were determined in accordance with the World Health Organization (WHO) criteria for lung tumors [30]. Written consent was obtained from all of the patients included in this study as recommended by French legislation.

### Tissue Microarray Manufacturing and Immunohistochemistry

For tissue microarray (TMA) manufacturing, representative areas of the NSCLC samples were identified by a surgical pathologist on hematoxylin-eosin (HE) stained slides. For each tumor, three core biopsies 1 mm in diameter were then taken from selected areas of donor blocks and arrayed into a recipient paraffin block using a manual tissue arrayer (Beecher instruments, Sun Prairie, USA). Sections (4 µm thick) of TMA blocks were cut and immunohistochemistry was performed using a rabbit polyclonal specific antibody raised against ID2 (ZMD.325, Invitrogen, Cergy Pontoise, France). Briefly, the TMA section was deparaffinized, rehydrated, and submitted to heat antigen retrieval in an ethylenediamine tetra acetic acid buffer (MS-Unmasker, pH 7.8, Diapath) for 20 min. ID2 antibody was applied at a 1/50 dilution. Immunostaining was performed with the Lab Vision Autostainer (Lab Vision, Fremont, USA) using a biotin-streptavidin-peroxidase detection system with diaminobenzidine as a chromogen (ChemMate™ Detection kit, DakoCytomation). Slides were hematoxylin counterstained. The TMA contained core samples of normal bronchiolar mucosa and lung tissue. Specificity of ID2 antibody was control by using purified ID2 protein as a negative control to block binding of ID2 antibodies to tumor cells (Figure S1).

Two independent observers (J.R., C.B.) evaluated both the localization and intensity of the ID2 staining. This evaluation was blinded to the clinical data. Both nuclear and cytoplasmic staining scores of tumor cells were established by multiplying the staining intensity scaled from 0 to 3 (null = 0, weak = 1, moderate = 2, and strong = 3) by the percentage of positive cells (0 = <10%; 1 = 10–25%; 2 = 26–50%; 3 = 51–75%; 4 = >75%). Global expression of ID2 in tumor cells was obtained by the addition of nuclear and cytoplasmic staining scores.

### Statistical Analysis

Multiple component analysis (MCA) was performed on data collected from 62 patients using SPAD software. This method is a descriptive multivariate analysis that allows for rapid identification of statistical relationships between variables. Briefly, the different categories, i.e., ID2 expression and clinicopathological features, were positioned in multidimensional space. In order to observe distances between points, they were projected on a plane of two orthogonal factors, *F*1 and *F*2. These ‘first’ factors were chosen to be the most representative of the global variance of the data. Because the position of categories of active variables (nuclear, cytoplasmic, and global expression of ID2) and the position of observations (stage, lymph node status, tumor status, histological types, grade of differentiation, genre, and smoking status) are calculated in the same way, they can be placed on the same graph (observations being replaced by gravity centers). DEMOD method (SPAD software), similar to the chi-square test, was also used to complement the MCA for analyzing the association between the active variables (nuclear, cytoplasmic, and global expression of ID2) and the illustrative variables (clinicopathological features).

Overall survival was calculated from the time of histology diagnosis to the date of death due to any cause. The overall survival rate was analyzed according to the Kaplan–Meier method, and the differences in survival rates were assessed by the log-rank test (Medcalc software version 7.3.0.1). Multivariate analysis of prognostic factors was performed using Cox proportional hazard model (Medcalc).

## Supporting Information

Figure S1 Immunohistochemical analysis of paraffin-embedded human NSCLC using rabbit polyclonal antibody against ID2. IHC was performed with antibody alone (A and C) or after neutralization of antibody by preincubation with 2 µg/mL of purified ID2 protein (ID2 recombinant protein P01, Abnova, Taiwan), overnight at 4°C, (B and D).
(9.11 MB TIF)
