# Supplementary figures and images for: The Intracellular Localization of ID2 Expression Has a Predictive Value in Non Small Cell Lung Cancer
Source: PLoS One. 2009 Jan 8;4(1):e4158. doi: 10.1371/journal.pone.0004158 (PMC2612745; doi:10.1371/journal.pone.0004158)

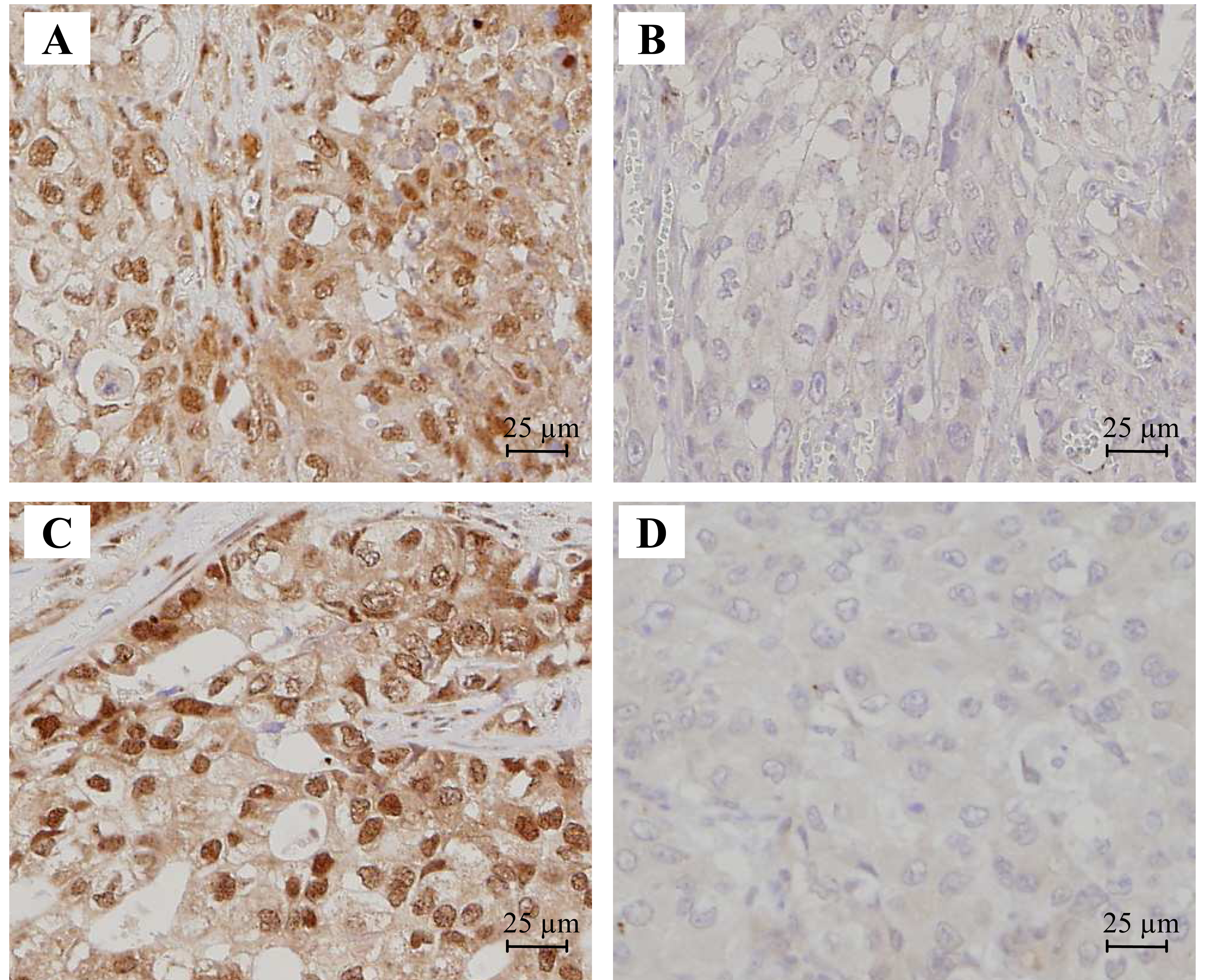

Supplement: Figure S1 — Immunohistochemical analysis of paraffin-embedded human NSCLC using rabbit polyclonal antibody against ID2. IHC was performed with antibody alone (A and C) or after neutralization of antibody by preincubation with 2 µg/mL of purified ID2 protein (ID2 recombinant protein P01, Abnova, Taiwan), overnight at 4°C, (B and D). (9.11 MB TIF) [file pone.0004158.s001.tif]
